# A longitudinal study of serological responses to *Coxiella burnetii* and shedding at kidding among intensively-managed goats supports early use of vaccines

**DOI:** 10.1186/s13567-017-0452-3

**Published:** 2017-09-15

**Authors:** Michael Muleme, Angus Campbell, John Stenos, Joanne M. Devlin, Gemma Vincent, Alexander Cameron, Stephen Graves, Colin R. Wilks, Simon Firestone

**Affiliations:** 1 Asia–Pacific Centre for Animal Health, Faculty of Veterinary and Agricultural Sciences, The University of Melbourne, Parkville, VIC 3010 Australia; 2 The Mackinnon Project, Faculty of Veterinary and Agricultural Sciences, The University of Melbourne, Werribee, VIC 3010 Australia; 3 Australian Rickettsial Reference Laboratory, Barwon Health, Geelong, VIC Australia

## Abstract

Vaccination against *Coxiella burnetii,* the cause of Q fever, is reportedly the only feasible strategy of eradicating infection in ruminant herds. Preventive vaccination of seronegative goats is more effective in reducing shedding of *C. burnetii* than vaccinating seropositive goats. The age at which goats born on heavily-contaminated farms first seroconvert to *C. burnetii* has not yet been documented. In a 16-month birth cohort study, the age at which goats seroconverted against *C. burnetii* was investigated; 95 goats were bled every 2 weeks and tested for antibodies against *C. burnetii*. Risk factors for seroconversion were explored and goats shedding *C. burnetii* were identified by testing vaginal swabs taken at the goats’ first kidding using a *com1* polymerase chain reaction assay. The first surge in the number of goats with IgM to *C. burnetii* was observed at week 9. Thus, a first vaccination not later than 8 weeks of age to control *C. burnetii* in highly contaminated environments is indicated. The odds of seroconversion were 2.0 times higher [95% confidence interval (CI) 1.2, 3.5] in kids born by does with serological evidence of recent infection (IgM seropositive) compared to kids born by IgM seronegative does, suggesting either in utero transmission or peri-parturient infection. The rate of seroconversion was 4.5 times higher (95% CI 2.1, 9.8) during than outside the kidding season, highlighting the risk posed by *C. burnetii* shed during kidding, even to goats outside the kidding herd. Shedding of *C. burnetii* at kidding was detected in 15 out of 41 goats infected before breeding.

**Electronic supplementary material:**

The online version of this article (doi:10.1186/s13567-017-0452-3) contains supplementary material, which is available to authorized users.

## Introduction

*Coxiella burnetii* causes Q fever in humans, a disease that manifests with influenza-like symptoms including fever and pneumonia in approximately 30% of those acutely infected [1, 2]. Chronic Q fever is characterised by debilitating arthritis, myopathy and cardiac malfunction [1–6]. Recent reports of chronic Q fever among individuals that had never been diagnosed with acute disease draws attention to the need for increased detection and treatment of sub-clinical Q fever infections [1, 2, 7]. The impact of the disease is further highlighted by increasing reports of chronic Q fever in children and mortalities of up to 13% among chronic Q fever patients [7–11]. These potentially severe impacts of Q fever underscore the need to implement stringent prevention and control measures. It is therefore imperative to have evidence of how effective the different control measures are in the control of *C. burnetii* infections.

Many large Q fever outbreaks have been linked to farms with small ruminants and key control strategies have targeted reducing shedding of *C. burnetii* by the animals [12–15]. For example, a ban on breeding, culling of pregnant animals and vaccination of animals before breeding were used to control the large Q fever outbreak in the Netherlands [12–15]. Additionally, vaccination against *C. burnetii* in animals was shown to be more effective in reducing the shedding of the organism when carried-out in seronegative animals than in seropositive ones, underscoring the need to vaccinate animals before they are infected with *C. burnetii* [16–18].

The age at which most animals born on infected farms first seroconvert to *C. burnetii* has not been documented. Several previously published studies point to the possibility that goats get infected early in life. For example, during a human Q fever outbreak that was linked to a goat farm in France, 52% of 3–4-month-old kids were reported to have been seropositive to *C. burnetii*; and approximately 90% of these were reported to have been infected with *C. burnetii* before they started kidding [19]. Similarly, 33% (192/589) of kid goats in another study undertaken on a farm linked to a human Q fever outbreak, were reported to have been infected before they started kidding and shed *C. burnetii* at their first kidding despite being kept away from adult goats as soon as they were born [17]. These studies do not indicate when the goats first seroconverted to *C. burnetii* although some of the results point to a time before 4 months of age.

Based on modelling of *C. burnetii* transmission and control in Dutch dairy farms, Bontje et al. found that vaccination of goats was the only control measure that could eradicate *C. burnetii* infections from infected herds [20]. The models estimated that it would take 7 years to eradicate the disease from infected farms if goats were to be vaccinated 1 month before breeding annually for all the 7 years [21]. There is a possibility that eradication of *C. burnetii* on infected farms could be achieved in shorter timeframes if vaccination of goats against *C. burnetii* were implemented at the age when most animals had not seroconverted. The Dutch transmission models supported the value of preventive vaccination over vaccination of already infected animals [20, 21]. Similarly, experimental and field studies that were carried out to assess the effectiveness of phase 1 vaccines administered prior to breeding mostly reported that vaccination only reduced the level of shedding at the herd level but did not prevent infection. This may be due to most of the goats being infected by the time of vaccination [16, 18, 19, 22].

This study therefore aimed to establish the age at which goats born on an intensively-managed goat dairy associated with human cases of Q fever first seroconverted to *C. burnetii* to provide recommendations on the timing of vaccination against *C. burnetii*. Additional objectives included: describing and comparing the nature (time of occurrence, duration and titres) of antibody-mediated immune responses to *C. burnetii* of goats that first seroconverted before the target breeding age (28 weeks) to those of goats that seroconverted later, and identifying risk factors and estimating any production losses associated with goats seroconverting before the target breeding age.

## Materials and methods

### Study design

In this longitudinal cohort study, 95 goats were followed from birth for 16 months. The animals were kept under routine animal management conditions on a large intensive dairy goat enterprise in Victoria, Australia. The enterprise holds approximately 5000 milking goats which are synchronised to concentrate kidding at four times each year (at March, June, September and November), in herds on five farms. The enterprise also has a flock of 1100 paddock-grazed extensively-managed dairy sheep. The sheep flock is kept separately from the goat flock. Since 2013, the enterprise was associated with 18 confirmed human cases of Q fever and tested polymerase chain reaction (PCR) positive for *C. burnetii* on various clinical and environmental samples [23]. Approximately 250 goats kid on each of the farms in every one of four kidding seasons, at a kidding rate of approximately two kids per goat. Only female kids and males from high producing animals (elite males) are kept for raising and production.

In herds infected with caprine arthritis and encephalitis virus (CAEV), some kids are routinely “snatch-reared”, i.e. removed from their does before they feed on colostrum to prevent transmission of CAEV. These were then bottle-fed pooled colostrum from a CAEV negative farm. The 95 goats (90 female and 5 elite males) in this study were on two of the farms (“GS” and “LC”), both of which were CAEV-positive herds that were previously shown to have a high prevalence of *C. burnetii* [24]. Therefore, the kids in this study were mostly snatch-reared and were bottle-fed colostrum (from the herd on a third farm, “FH”, holding CAEV-negative, but *C. burnetii*- positive goats) within 8 h after birth. The colostrum is routinely heat-treated before it is fed to the goats. It is anticipated that heat-treatment would not lower the quality of immunoglobulins in the colostrum. The kid goats were then fed a milk replacer and weaned at approximately 10 weeks of age. At 28 weeks of age (target breeding age), the female goats that weighed ≥ 23 kg were mated using adult elite males (not the elite males followed in this study). The goats are routinely weighed at weaning and before breeding.

### Sample size

The minimum required sample size was estimated to be 40 kids to have 80% power of detecting a statistically significant difference between paired samplings (day 0–2 weeks sampling as well as 0–12 weeks) assuming seroconversion to *C. burnetii* would be very rare in kids between 0 and 2 weeks of age (< 1%) and that 20% of the kids would have seroconverted by 12 weeks of age; at a 5% chance of a type I error. Based on this sample size calculation, with consideration of anticipated mortality, losses to follow-up and multivariable effects, 95 goats were recruited from two kidding seasons; 33 in March 2014 kidding season (cohort 1) and 62 in June 2014 kidding season (cohort 2). Goats in cohort 1 were recruited from farms GS and LC while goats from cohort 2 were recruited only from LC. All animal sampling was conducted with the approval of the University of Melbourne Animal Ethics Committee, Application Number 1413118.

### Examination of antibody-mediated immune responses to *C. burnetii* in goats from birth to 16 months of age

To investigate the age at which goats seroconvert to *C. burnetii* as well as to describe and compare antibody-mediated immune responses to *C. burnetii* among goats that seroconverted before and after the target breeding age, blood samples were taken from the recruited goats at birth (before feeding colostrum), then every 2 weeks for the first 7 months of life and then every 4 weeks until the end of the study, and tested for serum IgM and IgG antibodies against *C. burnetii* antigens using a previously validated indirect immunofluorescence assay (IFA) [24].

In this study, the IFA applied was on a dilution series commencing at 1:160. As described in detail elsewhere [24], at this cut-off there was minimal background fluorescence in negative control goat samples obtained from New Zealand, an OIE-declared *C. burnetii* free country. Appling a 1:160 cut-off, the IFA has also been shown to be highly sensitive for IgG (94.8%) and IgM antibodies (88.8%) [24]. The repeatability of the IFA was previously estimated to be 100% for IgG phase 1, 96.9% for IgG phase 2 and 78.1% for both IgM phase 2 and IgM phase 1 using samples re-tested after 3 months of storage at 4 °C [24]. The IFA slides were read by two experienced technicians with 94.4% agreement (Cohen’s *Kappa* = 0.88) [24].

To quantify serum antibodies to *C. burnetii* two-fold serial dilutions of sera were prepared, from 1:160 (the previously validated cut-off value) to 1:1280, and tested for IgM and IgG antibodies against *C. burnetii* antigen using the IFA [24]. The testing of the twofold serial dilutions from 1:160 to 1:1280 was undertaken on sera from 54 of the 95 goats that were present throughout the first 12 months of the study. Among those excluded were animals that were lost to follow up, including those that died in the first 10 weeks of the study before immune responses were mounted. Repeat samples from each goat were also used to describe the duration of the immune response and the proportion of goats mounting secondary immune responses (immune responses that occur after the initial antibody response had declined). All samples were tested in duplicate for IgM and IgG against phase 1 and 2 *C. burnetii*.

Colostrum antibodies were identified through the occurrence of high IgG phase 1 titres in kid goats that were previously seronegative but seroconverted after feeding colostrum, as IgG antibodies occur late in the course of infection and are not expected to occur within 2 weeks in goats that had been negative. After the decay of IgG phase 1 maternal antibodies, new *C. burnetii* infections were considered to have occurred when serum IgM followed by IgG antibodies against phase 2 antigens were identified in previously seronegative goats or a twofold rise in antibody titre was detected following seroconversion to IgG phase 2, as is routine practice in human and veterinary *C. burnetii* diagnostics [25, 26]. Antibodies against the phase 2 antigens of *C. burnetii* appear early in the course of infection while antibodies against phase 1 antigens appear much later in the course of infection [25, 26]. Incidence rates were estimated using an animal time at risk denominator calculated as the number of weeks from birth to the sampling time immediately before the first seroconversion (from negative to positive for both IgM and IgG or ≥ twofold rise in IgG titre against phase 2) or loss to follow-up of each animal. Half of the time between the last negative sample and the first positive sample or loss to follow up was added to the length of time an animal was considered at risk [27].

Pooled colostrum collected from the CAEV-negative farm, FH, was tested for *C. burnetii* IgG antibodies to phases 1 and 2 *C. burnetii* using the *IDEXX* commercial ELISA kit validated for detecting antibodies in milk whey at a 1:5 dilution [24, 28]. Undiluted whey from the pooled colostrum was also tested for IgG and IgM antibodies against phase 1 and 2 *C. burnetii* using the IFA protocol that had been validated for use with goat serum, to ascertain the type of antibodies the goats obtained from colostrum, as described previously [24, 28]. The colostrum was also tested for *C. burnetii* DNA using a DNA extraction protocol and PCR assay targeting the *com 1* gene of *C. burnetii* as previously described [24, 29].

### Identification of risk factors associated with seroconversion to *C. burnetii* before breeding

The probability of remaining seronegative at the different sampling points until the target breeding age was estimated using survival analysis. Initially, three parametric models (namely the Weibull, the Cox proportional hazard model and the Exponential model) were constructed to estimate the probability of goats remaining seronegative from birth to breeding. These were then analysed for goodness of fit based on the Akaike information criterion (AIC) [30]. As the Weibull model displayed the best fit to the data, the probability density function of the time to first seroconversion (change from negative to positive for both IgM and IgG or ≥ twofold rise in IgG titre against phase 2) (*t*) was derived from the Weibull distribution using the equation:

$$f(t) = \alpha \lambda t^{\alpha - 1} e^{{\lambda t^{\alpha } }}$$

where *f(t)* is the probability density function of seroconversion at the different time points *t*; λ > 0 is the probability of seroconversion or scale parameter and α > 0 is the shape parameter. α is 1/σ, σ being a variance-like parameter on the log-time scale. From this the survival (i.e. proportion yet to seroconvert at each timepoint), *S*(*t*), was derived as:

$$S\left( t \right) = e^{ - \lambda t\alpha }$$

The influence of qualitative risk factors including farm, cohort, *C. burnetii* exposure status of the source does, failure of passive transfer and the quality of passive transfer, on seroconversion was tested using the Weibull survival model. The *C. burnetii* exposure status of the source does was obtained by testing serum samples from the source does for IgG and IgM antibodies against *C. burnetii* at the time of birth of the study kid-goats. The occurrence of passive transfer was inferenced through the detection of IgG phase 1 maternally-derived antibodies in the study goats at 2 weeks of age while the quality of passive transfer was assessed using the titre of maternally-derived IgG phase 1 and 2 antibodies detected the kid-goats at 2 weeks of age as well as the detection of IgG phase 1 maternally-derived antibodies in kid goat sera after 4 weeks of age. The risk factors were included in separate univariable Weibull models to assess their influence on the probability of goats remaining seronegative or seroconverting at various times before the target breeding age. The univariable Weibull models testing the influence of risk factors on the probability of seroconversion is described by the equation:

$$\lambda_{i} = e^{{ - (\mu + \beta x_{j} )/\sigma }}$$

where the influence of risk factor *x*_*j*_, for the *i* ^*th*^ goat is modelled through the seroconversion probability parameter λ_j_ and $$\alpha = {\raise0.7ex\hbox{${1 }$} \!\mathord{\left/ {\vphantom {{1 } \sigma }}\right.\kern-0pt} \!\lower0.7ex\hbox{$\sigma $}}$$.

Kaplan–Meier survival curves and univariable Weibull regression coefficients were then generated for different factors at cohort, farm (farm of birth), doe (IgG and IgM serological status) and individual kid level (level and duration of antibodies to *C. burnetii* derived from colostrum) and used to assess how the different risk factors influence seroconversion. Multivariable Weibull regression models were constructed (based on appropriate fit to the data) including all factors statistically significant in univariable analysis at *p* < 0.2. The coefficients of the Weibull model were converted to hazard ratios using the *ConvertWeibull* package in R version 3.1.3 [31, 32], and only variables associated with the relevant outcome at *p* < 0.05 were retained in final models. The residuals and predicted values of the variables in the final models were analyzed and scaled-schoenfied residual plots were prepared for the variables in the final models.

To ascertain whether exposure to kidding periods resulted in increased numbers of infected goats, the rate of occurrences of IgM or IgG responses against *C. burnetii* during and outside the kidding season was compared using the animal time at risk denominator and the number of IgM and IgG responses as the numerator in the “compare 2 rates” function in Open Epi, version 3.01. The number of IgG and IgM responses were counted whenever a change from negative to positive to either IgM or IgG occurred during testing at the screening dilution. Primary and secondary IgM or IgG responses were considered to have occurred during the kidding period if they were detected within or 15 d after the kidding months. The extra 15 d were included to cater for the time required for antibody-mediated immune responses to occur in case goats became infected towards the end of the kidding season. Antibody-mediated immune responses that could have resulted from infections due to *C. burnetii* shed at either early kidding or late-term abortions, occurring within 2 weeks prior to the start of the kidding season, would fall within the kidding months and thus catered for in the “within kidding” counts of seroconversion. Only antibody-mediated immune responses that occurred after weaning (week 10) were included in this analysis as they were considered to be immune responses arising from environmental exposure to *C. burnetii* and not infection as neonates during the kidding process.

The period between any two consecutive sampling points from week 10 to the end of the study were classified as occurring “within” or “outside” the kidding season based on whether or not they fell during the kidding month and 15 d later. Animal time at risk “within” and “outside” the kidding period was computed for each animal as the number of weeks between the two consecutive sampling points if its samples tested negative at both points; half of the length of time between two sampling points if antibody-specific immune responses occurred (from negative to positive at 1:160 screening dilution), seroreversion (from a positive to either IgM or IgG at the 1:160 screening dilution to negative) or loss to follow-up occurred. For periods with one sampling point falling within the kidding season and the other outside the kidding season, the animal time at risk was divided equally between the within and outside kidding categories.

### Estimating the effect of pre-breeding seroconversions on shedding of *C. burnetii* at kidding and on production parameters

To estimate the proportion of shedding arising from goats that seroconverted before the target breeding age, vaginal swabs were obtained within 24 h of the goats’ first kidding and tested using the real genomics DNA extraction protocol (Real Biotech Company) and a PCR targeting the *com1* gene of *C. burnetii* [29]. The difference in the risk of delayed kidding (not kidding by 12 months of age), delayed joining (not bred at 7 months) and shedding of *C. burnetii* among goats that seroconverted before the target breeding age and those that seroconverted after the target breeding age was estimated using two-by-two contingency tables, and comparing relative risks with estimation of 95% confidence intervals and Fisher’s exact test statistic in STATA.

The association between seroconversion before breeding and outcomes of weight at weaning, and weight change between weaning and breeding, were estimated using multivariable linear regression models, and adjusting for cohort and farm, using STATA 13.0. The model residuals and the weight variables used were also analysed for normality in STATA 13.0.

## Results

### Antibody-mediated immune responses to *C. burnetii* in goats from birth to 16 months

**Figure 1:**
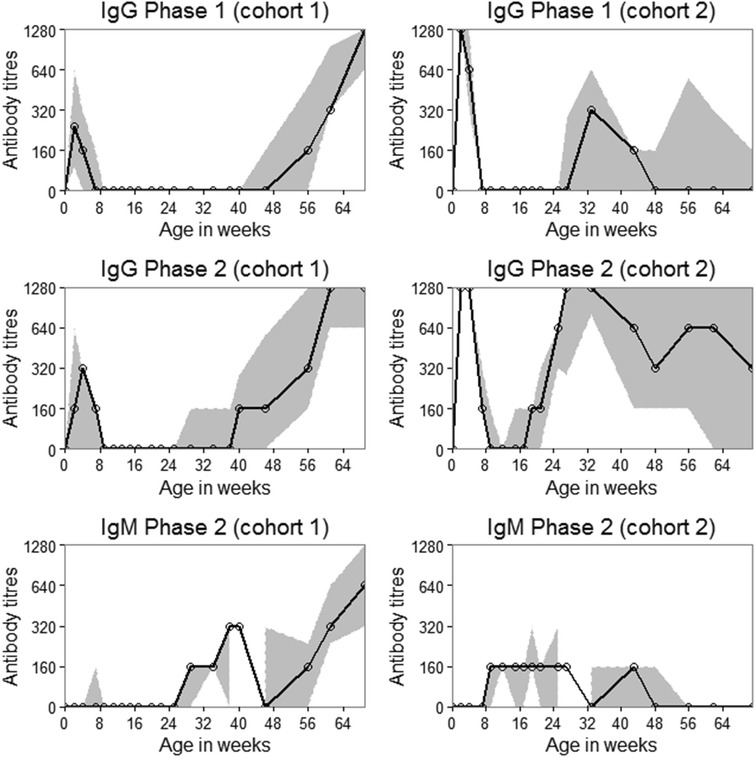
**Median and inter-quartile range of antibody titres against** ***C. burnetii*** **in intensively-managed goats**. High IgG titres against phase 1 and phase 2 were detected after feeding colostrum during the follow up of 95 kid-goats. IgG phase 1 antibodies against *C. burnetii* were used to differentiate maternally-derived antibodies from immune response to *C. burnetii* exposure; IgG antibodies against phase 1 waned by 7 weeks. IgM titres started to rise from 2 weeks of age and reached the first peak at 8 weeks of age. This was followed by a rise in IgG phase 2 titres. A rise in IgG phase 1 titres then followed at 24 weeks of age in cohort 2 and 40 weeks in cohort 1. The solid light grey band shows the inter-quartile range of titres while the solid black line shows the median titres.

No antibodies *against C. burnetii* were detected before goats were fed colostrum (Figure 1). High IgG antibody titres to phase 1 and phase 2 *C. burnetii* were detected after feeding colostrum (Figure 1). IgG antibodies against phase 1 *C. burnetii* were used to differentiate maternally-derived antibodies from responses to *C. burnetii* exposure; IgG antibodies against phase 1 waned by week 7 (median duration of presumably-colostrum derived antibodies: 7 weeks; inter-quartile range: 4–7 weeks). Of the 80 goats sampled after they had been fed colostrum, 71 (89%) became seropositive for IgG to phase 1 antibodies (presumably maternally-derived). Furthermore, all these, except one, were also positive for IgG antibodies against phase 2 *C. burnetii*. Colostrum was tested and found to have IgG antibodies to phase 1 and 2 *C. burnetii* antigens using ELISA (optical density [OD] of 1.09 at 0.40 cut-off) and IFA (titres of 1024 IgG to phase 1, 1024 IgG phase 2) and contained negligible amounts of IgM against phase 2 (IFA titre = 40) and no IgM to phase 1 *C. burnetii*.

**Figure 2:**
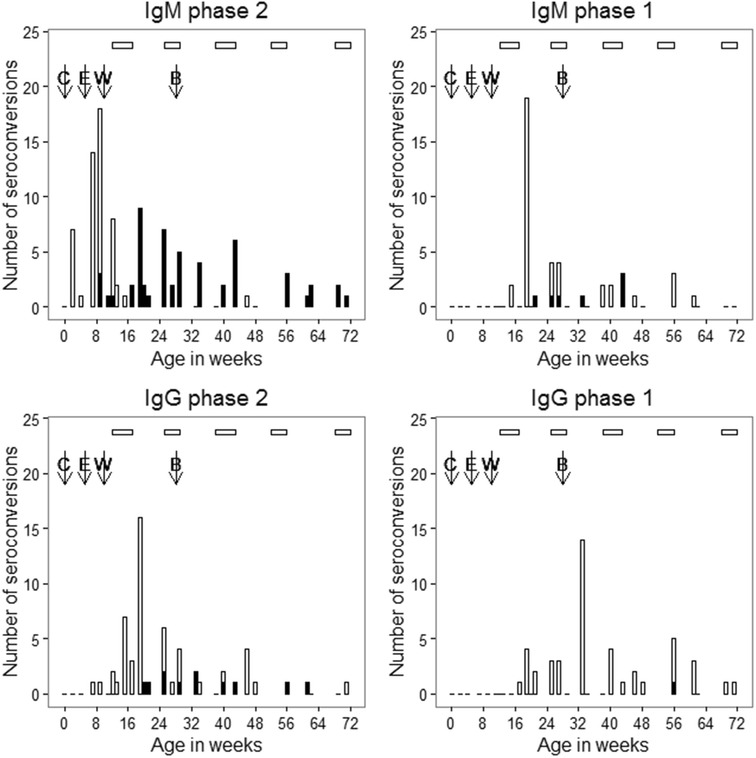
**Occurrence of seroconversions against** ***C. burnetii*** **during a longitudinal study in intensively-reared goats.** The white vertical bars represent primary immune response to presumed first exposure in goats. The primary immune responses occurred soon after IgG phase 1 colostrum-derived antibodies had waned; they were the first immune responses detected in goats that had no IgG phase 1 colostrum-derived antibodies. The vertical grey bars represent secondary seroconversions mounted following seroreversion from primary seroconversions. The horizontal white bars on the top of the graph represent the kidding seasons. Colostrum was fed at birth (C) and (E) is the median point at which IgG phase 1 and 2 antibodies detected soon after feeding colostrum waned. (W) corresponds to the target weaning age while (B) is the target breeding age.

IgM seroconversion to phase 2 *C. burnetii* occurred as early as week 2 after birth; however, the first surge in the number of goats that seroconverted to phase 2 *C. burnetii* was observed at 9 weeks of age (Figure 2). Similarly, IgM titres to phase 2 *C. burnetii* rose as early as 2 weeks into the study, but the first peak was observed at 9 weeks of age as shown in Figure 1. This was followed by a rise in IgG titres to phase 2 *C. burnetii* and a rise in IgG titres to phase 1 *C. burnetii* at 24 and 40 weeks of age in cohort 2 and cohort 1, respectively (Figure 1).

From birth to 10 weeks, 40 out of the 80 goats (50%) present at the second sampling point showed IgM seroconversion to phase 2 *C. burnetii* while between week 10 and week 28, another 17 out of the 76 (22%) goats present at week 10 showed IgM seroconversion to phase 2. From birth to week 10, IgG seroconversion to phase 2 was detected in 2 out of the 80 goats (3%) and between week 10 and week 28, a total of 38 out of the 76 goats (50%) showed IgG seroconversion to phase 2. One goat had IgG antibodies against phase 2 without a detectable IgM response against phase 2.

Nine goats did not have detectable IgG antibodies to phase 1 after feeding colostrum. Of these, seven goats showed IgM seroconversion to phase 2 by week 28 (three at week 2, three at week 7 and one at week 25) and only one seroconverted after week 28. One of the nine goats that did not have IgG to phase 1 after feeding colostrum died at week 4 before seroconverting to *C. burnetii*.

A total of 19 out of the 95 goats died by week 5, before any seroconversion was observed. Of these, only three had the initial IgG to phase 1 and 2 observed after feeding colostrum while one goat did not have IgG antibodies to phase 1 after feeding colostrum. The rest of these goats (15) died before the second sampling, thus their serological status after feeding colostrum could not be assessed. Another six goats died between week 5 and week 10, all of which had detectable IgG to phase 1 after feeding colostrum. One of these showed IgM seroconversions to phase 2. An additional eight deaths occurred between week 10 and week 28, all except one had IgG antibodies against phase 1 after feeding colostrum and only three had seroconverted with IgM against phase 2.

The crude incidence rate was seven seroconversions (95% CI 5.7, 8.9) per 100 goat weeks at risk. Equivalent interpretations include seven seroconversions per 100 goats at risk for 1 week, or per 50 goats followed for 2 weeks. Whilst there was no statistically significant difference in the rates when compared across time periods (from birth to weaning, weaning to target breeding age and after the target breeding age), see Table 1, there was a trend suggesting gradually increasing risk over time.

**Table 1:** **The incidence rate of seroconversion to** ***C. burnetii*** **before and after the breeding age**.

Time period (weeks)	Number at risk	Goat weeks at risk	Number of seroconversions	Incidence rate (per 100 goat weeks at risk)	Incidence rate ratio (95% CI)
0–10	95	618.5	40	6.5 (4.6, 8.8)	1.00 (reference)
11–28	32	238.0	18	7.6 (4.5, 12.0)	1.17 (0.67, 2.04)
29–71	8	61.5	8	13.0 (5.6, 25.6)	2.01 (0.94, 4.30)
Total	135	918.0	64	7.0 (5.7, 8.9)	–

10 weeks is the target weaning age, 28 weeks was the target breeding age in the intensively-managed goats on the study farm in Victoria, Australia, 2014–2015

*CI* confidence interval

Secondary IgM against phase 2 was detected in 42 out of the 70 goats (60%) present at the time of weaning. A total of 24 of these secondary IgM immune responses occurred before the target breeding age and the other 18 occurred after the target breeding age. Secondary IgG immune responses against phase 2 were detected in only 11 goats, four of these occurring before the target breeding age. Only seven goats had a secondary IgM response against phase 1 and only one goat had a secondary IgG response against phase 1.

**Figure 3:**
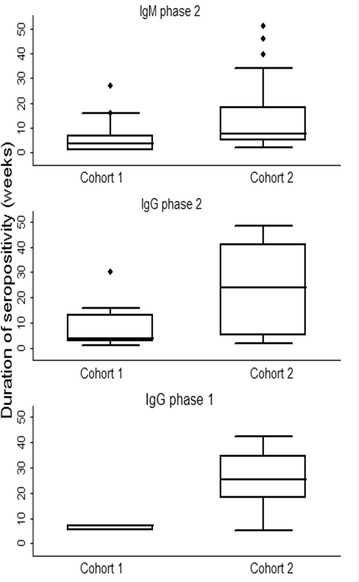
**Box plots of the duration of IgG and IgM antibodies against** ***C. burnetii*** **in intensively-reared goats.** Overall the duration of antibodies to *C. burnetii* was higher in cohort 2 compared to cohort 1.

The duration of IgG and IgM immune responses are described in Table 2. The duration of primary immune responses was significantly higher in cohort 2 than in cohort 1, except for IgM against phase 2 as shown in Figure 3. Overall, immune responses that occurred before the target breeding age were not statistically different from those that occurred after the target breeding age (Table 2).

**Table 2:** **Comparison of the duration of pre-breeding to post-breeding antibody responses to** ***C. burnetii*** **in goats**.

Antibody type	Type of immune response	Time of occurrence	Duration of antibody responses in weeks			
			n (rev)	Mean	Median (range)	*p* value*
IgM phase 2	Primary	Pre-breeding	57 (55)	10.9	5.5 (1.0, 51.0)	0.570
		Post-breeding	7 (7)	5.9	6.0 (2.0, 12.0)	
	Secondary	Pre-breeding	24 (24)	10.4	9.0 (1.0, 34.0)	0.523
		Post-breeding	18 (18)	8.4	4.0 (2.0, 33.0)	
IgG phase 2	Primary	Pre-breeding	40 (27)	19.4	12.0 (1.0, 48.5)	0.376
		Post-breeding	14 (5)	7.5	6.0 (2.5, 13.0)	
	Secondary	Pre-breeding	4 (3)	30.7	42.5 (3.0, 46.5)	0.564
		Post-breeding	7 (2)	25.8	25.8 (17.0, 34.5)	
IgM phase 1	Primary	Pre-breeding	29 (29)	5.1	4.0 (1.0, 12.0)	0.197
		Post-breeding	9 (7)	2.9	3.0 (1.0, 6.0)	
	Secondary	Pre-breeding	3 (3)	1.3	1.0 (1.0, 2.0)	0.127
		Post-breeding	4 (4)	2.9	3.5 (1.0, 3.5)	
IgG phase 1	Primary	Pre-breeding	40 (12)	23.7	22.5 (5.0, 42.5)	0.897
		Post-breeding	14 (14)	20.5	22.3 (5.0, 34.5)	
	Secondary	Pre-breeding	0 (0)	–	–	–
		Post-breeding	1 (1)	11.5	–	

Pre-breeding responses are those that started before the target breeding age while post-breeding responses are those that started after the target breeding age in intensively managed goats on the study farm in Victoria, Australia, 2014–2015

*rev.* number of antibody responses that seroreverted (moved from positive to negative) which were used in calculating the duration of serological positivity, *n* total number of antibody responses

* p values derived using the Mann–Whitney U test comparing the duration of pre-breeding immune responses to the post-breeding immune responses

### Risk factors associated with seroconversion to *C. burnetii* before breeding

**Figure 4:**
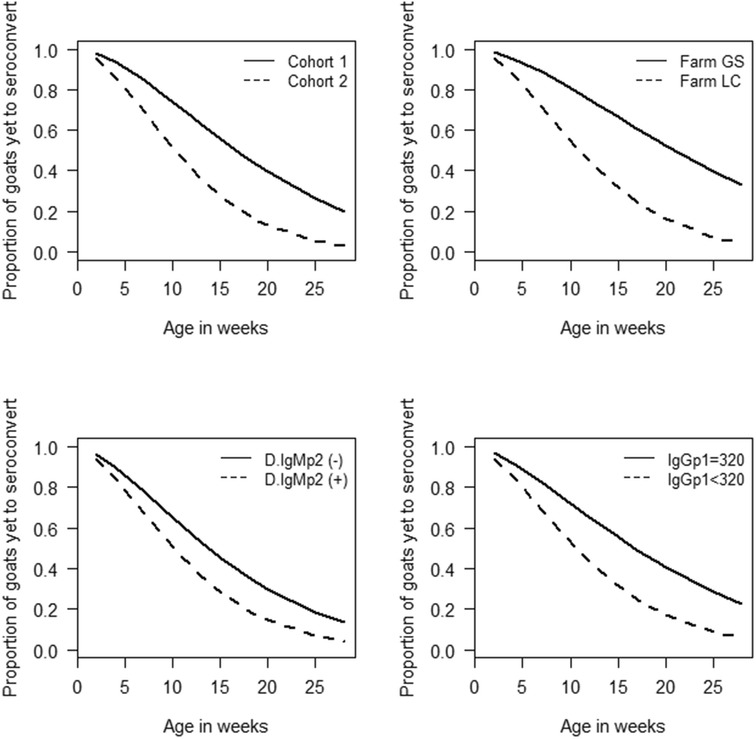
**Weibull survival curves comparing the pre-breeding probability of remaining seronegative to** ***C. burnetii*** **in goats.** D.IgMp2 = IgM serological status of the source does with (D.IgMp2(−) representing negative does and (D.IgMp2(+) the positive ones. IgGp1 =colostrum derived antibody titres dichotomised at a cut-off of 320 (IgGp1). Goats in cohort 1, farm GS and those born by (D.IgMp2(−) as well as those with IgGp1 =320 had statistically significantly (*p* < 0.1) higher probabilities of remaining seronegative before breeding than goats from cohort 2, farm LC as well as those from D.IgMp2(+) and those with IgGp1 < 320 colostrum antibodies.

The probability of goats remaining seronegative to *C. burnetii* for the first 28 weeks of life was 16.7% (95% CI 9.4, 29.4) (Additional file 1). The Weibull model had the best fit for these data with (AIC = 440.9) compared to the Cox proportional hazards model (AIC = 457.5) and the Exponential model (AIC = 460.6). Being from farm GS, being a member of cohort 1, having colostrum-derived IgG phase 1 antibodies titres ≥ 320 and being born by an IgM seronegative doe were all associated with a higher probability of not seroconverting against *C. burnetii* before 28 weeks of age on univariate analysis as shown in Figure 4. Outputs of multivariable Weibull regression are presented in Table 3, and for completeness, univariable results are presented in Additional file 2. The odds of seroconversion were 2.0 times higher [95% confidence interval (CI) 1.2, 3.5] in kids born by does with serological evidence of recent infection (IgM seropositive) compared to kids born by IgM seronegative does. Model residual analysis showed that the assumptions for the Weibull model were not violated.

**Table 3:** **Multivariable Weibull accelerated failure-time regression model assessing risk factors for seroconversion to** ***C. burnetii*** **before breeding**.

Variable	Levels	n	Serocon.	Coef.	SE (Coef.)	p value	Survival rate ratio (95% CI)	Hazard ratio (95% CI)
Cohort	Cohort 2	62	42	−0.60	0.11	0.001	0.55 (0.39, 0.78)	2.73 (1.45, 5.16)
	Cohort 1	33	16	0.00 (ref)			1.00	1.00
Doe IgM	Positive	37	29	−0.42	0.16	0.008	0.65 (0.48, 0.89)	2.04 (1.19, 3.54)
	Negative	56	28	0.00 (ref)			1.00	1.00
Intercept	–	–	–	3.89	0.35	< 0.001	–	–

In intensively-reared goats in Victoria, Australia, 2014–2015. Interpretation: After adjusting for the effect of cohort, kids born by does that had positive IgM titres (Doe IgM = positive; indicating recent exposure) were 2.04 times more likely to seroconvert within the first 6 months of life compared to those born by IgM seronegative does. Log likelihood = −201.1. *Coef*. coefficient. Similarly, after adjusting for the effect of farm, kids born by does that had positive IgM titres were 2.23 times (95% CI 1.29, 3.86) more likely to seroconvert within the first 6 months of life compared to those born by IgM seronegative does; see Additional file 3

The rate of occurrence of IgG immune responses to *C. burnetii* was 4.5 times higher (95% CI 2.1, 9.8) within the kidding season than outside the kidding season among goats in cohort 2 (Additional file 4), whereas, the rate of occurrence of IgG immune responses was comparable within and outside the kidding season among goats in cohort 1.

### The effect of pre-breeding seroconversions on the shedding of *C. burnetii* and on production parameters

Overall, 18 out of 46 goats were detected as shedding *C. burnetii* by PCR at their first kidding. Shedding was detected in 15 out of the 41 goats that seroconverted before week 28, some of which remained seropositive until kidding (Additional file 5). Out of the five goats that had not seroconverted by week 28, three goats had detectable *C. burnetii* DNA in vaginal swabs taken at their first kidding.

The proportion of goats that were joined on time was comparable in goats that seroconverted before 28 weeks (28/50 goats, 56.0%) and those that had not seroconverted by week 28 (6/8 goats, 75.0%). Similarly, the proportion of goats kidding on time was comparable in goats that seroconverted before week 28 (19/50 goats, 38.0%) and those that had not seroconverted by week 28 (5/8 goats, 62.5%).

The multivariable linear regression model showed no statistically significant difference in pre-breeding weight between goats that seroconverted early and those that seroconverted later (Table 4). At weaning, goats in cohort 2 weighed 4.8 kg less than goats in cohort 1 (95% CI 6.6, 3.1 kg less); and 30 out of the 49 goats (61%) with weaning weight records in cohort 2 had seroconverted to either IgG or IgM phase 2 by week 10 while only 5 out of the 13 of goats (39%) with weaning weight data in cohort 1 had seroconverted by week 10. Between the time of weaning and the target breeding age, goats in cohort 2 gained 5.3 kg more than goats in cohort 1 (95% CI 2.4, 8.1). Only 10 out of the 38 goats (26%) in cohort 2 seroconverted between weaning and the target breeding age compared to 6 out of the 12 goats (50%) in cohort 1. The distribution of the model residues and weight variables used in the model were within acceptable limits (skewedness ± 1).

**Table 4:** **Multivariable linear regression analysis of the effect of seroconversions to** ***C. burnetii*** **on goat weights**.

Model	Variable	Levels	n	Coef.	SE (coef.)	*p* value	95% CI of Coef.
Effect of seroconversion on weight at weaning^a^	Cohort	Cohort 2	49	−4.84	0.88	< 0.001	−6.61, −3.08
		Cohort 1	13	0.00 (ref)			
	Sex	Male	4	3.64	1.46	0.016	0.71, 6.56
		Female	58	0.00 (ref)			
	Time of first seroconversion	After 28 weeks	10	−0.33	1.00	0.740	−1.03, 2.35
		10–28 weeks	17	0.66	0.84	0.435	−2.36, 1.66
		0–10 weeks	35	0.00 (ref)			
	Intercept	–	–	17.71	0.89	< 0.001	15.93, 19.48
Effect of seroconversion on weaning to breeding weight change^b^	Cohort	Cohort 2	38	5.27	1.41	0.001	2.43, 8.11
		Cohort 1	12	0.00 (ref)			
	Time of first seroconversion	After 28 weeks	3	0.98	2.52	0.700	−4.09, 6.04
		10–28 weeks	16	−0.60	1.31	0.652	−3.23, 2.04
		0–10 weeks	31	0.00 (ref)			
	Intercept	–	–	7.67	1.40	< 0.001	4.86, 10.49

Interpretation: ^a^ No statistically significant difference in weaning weight was observed between goats that seroconverted before breeding and those that seroconverted post-breeding after adjusting for cohort and sex. However, goats in cohort 2 had weighed 4.84 kg lower at weaning than goats in cohort 1

^b^Similarly, no statistically significant difference in weaning-breeding weight change was observed between goats that seroconverted before breeding and those that seroconverted post-breeding after adjusting for cohort. Surprisingly, goats in cohort 2 weighed 5.27 kg more than goats that had not seroconverted by breeding. Sex was not included in model^b^ because all male animals were lost from the study by breeding time

## Discussion

To the best of our knowledge, this is the first birth cohort study to systematically investigate *C. burnetii* seroconversions in goats on infected farms over an extended period from birth. Furthermore, this study was undertaken in the absence of vaccination of goats or other control measures that would reduce the shedding of *C. burnetii* in infected goats. Therefore, the patterns of seroconversion or transmission described in this study can be considered representative of transmission of *C. burnetii* among young goats in a heavily contaminated intensive dairy herd.

Detection of antibodies in serum was considered the most effective way of detecting recent infection with *C. burnetii* in goats before breeding. Substantial quantities of *C. burnetii* are shed in milk, vaginal mucus and faeces around parturition due to massive replication of *C. burnetii* in the placenta [33–35]. However, shedding is only detectable over a very short timeframe and not before breeding [33–35]. Additionally, *C. burnetii* is present in low concentrations in blood, and for only a few days following infection, unlike antibodies which appear 1–3 weeks after infection and typically last for weeks to months depending on antibody class [26].

High proportions of goats had seroconverted to IgM and IgG against phase 2 *C. burnetii* by week 28 of the study which indicates that majority of the goats born on *C. burnetii* positive farms are infected early in life. Seroconversions were observed well before the goats were mated, which is contrary to the notion that trophoblasts are required for establishment of *C. burnetii* infection in ruminants. Infection of young non-pregnant goats with *C. burnetii* has been disputed in previous studies [17, 19, 36–39]. Although some studies suggest that *C. burnetii* requires trophoblasts for successful establishment of infection in ruminants [35, 36, 40, 41], we are not aware of any study that has investigated whether the absence of trophoblasts in young and male animals prevented the establishment of *C. burnetii* infection. Previous work has suggested that *C. burnetii* infections require trophoblasts to infect ruminants. This was hypothesised after observing primary replication and histopathological lesions only in trophoblasts following experimental infection of pregnant goats with *C. burnetii* [36]; that finding has been referenced in other studies [20]. However, it is possible that the organisms could have been present in other tissues in quantities undetectable by the methods used, as noted by the authors of the study [36].

Despite some studies suggesting that trophoblasts are required for establishing infection in ruminants, a number of studies investigating other aspects of *C. burnetii* on infected farms have reported a high proportion (> 50%) of seroconversions to *C. burnetii* in young ruminants before breeding [17, 19], which is similar to the level detected in our study. Another study reported a seroprevalence of 9.8% in 6-month-old goats that had been kept in a closed facility with no exposure to adult goats [39]. In some instances, young animals were thought not to pose much risk in the transmission of *C. burnetii* thus excluding them from *C. burnetii* studies [16]. It is likely that the discrepancy in the proportion of young animals that seroconvert to *C. burnetii* among several studies [17, 19, 39] is highly dependent on the proportion of parent does shedding *C. burnetii* and the timing of the samplings relative to the progression of the outbreak. The proportion of adult goats shedding *C. burnetii* is likely to increase over time in an uncontrolled outbreak thus increasing the dose of *C. burnetii* organisms each susceptible animal in the herd is exposed to; and henceforth increasing the number of animals infected with *C. burnetii*. It is not clear if the build-up in the proportion of goats shedding *C. burnetii* would be affected by development of immunity after repeated exposure to *C. burnetii* but one study reported that infected goats shed *C. burnetii* for at least two successive kiddings [34].

Furthermore, studies that detected *C. burnetii* organisms in spleens, liver, lungs, kidneys and hearts of young goats [18, 36, 37, 42] as well as interstitial non-suppurative pneumonia and granulomatous hepatitis lesions, similar to those observed in adult goats infected with *C. burnetii,* adds further evidence to the notion that goats can be infected early in life [42]. In most of these studies, it is apparent that diagnosis was made on a few tissues that were submitted, and the majority of the tissues were from kids that died perinatally as well as those that had been sacrificed after birth.

These studies provide useful clues to potential sites of replication and persistence of *C. burnetii* infection in non-pregnant young goats that could possibly result in shedding of low levels of the bacterium before breeding. However, the site of persistent *C. burnetii* infection in young ruminants is still unknown, unlike in adult goats where *C. burnetii* infection persists in the mammary glands and uterus resulting in continuous shedding of *C. burnetii* in milk long after kidding [43, 44]. The site of persistent infection in young animals needs to be investigated, notwithstanding the possibility that the organism may be present in low numbers below the detection limit of most diagnostic tools.

The initial rise in IgG phase 1 and IgG phase 2 following colostrum feeding is probably due to uptake of antibodies against *C. burnettii* in colostrum. This is further supported by the high concentrations of IgG specific for phase 1 and phase 2 as well as negligible quantities of IgM that were detected in colostrum. The IgG and IgM concentrations in colostrum reported by this study are in accordance with another study that reported colostrum IgG concentrations of 41.2 mg/mL and IgM concentrations of 1.9 mg/mL [45]. In the event of infection at birth or around the time of feeding colostrum, IgG antibodies to phase 1 against such infections would not be detectable within 2 weeks as IgG phase 1 antibodies appear much later in the course of infection [26, 36, 46, 47]. Thus, the extremely high IgG phase 1 and phase 2 antibody titres (Figure 2) observed in the serum of kids at 2 weeks were most likely derived from colostrum.

Early IgM responses were detected mostly in kids that did not receive sufficient colostrum. This suggests that antibodies and other immune components in colostrum are protective against *C. burnetii* infection. This further explains the surge in IgM and IgG immune responses after the IgG phase 1 colostrum antibodies (antibodies detected immediately after feeding colostrum) wane, around week 7 (median), and thus suggests that goats are at risk after the depletion of colostrum antibodies. These findings also suggest that, in this study, ingestion of colostrum was protective in goat kids.

Colostrum could also have been a source of *C. burnetii* infection although this appears not to have been the case in this study possibly due to heat treatment of colostrum before it is fed to the goats. In any case, it appears that infection resulting from ingestion of contaminated milk is not readily achieved and observations in humans ingesting contaminated milk failed to demonstrate resulting infection [48, 49]. It is therefore worthwhile to ensure that all goats are fed enough colostrum containing antibodies against *C. burnetii*, especially in instances where new-born goats are snatched from their mothers before they ingest colostrum. Perhaps, efforts could also be aimed towards increasing antibodies and immune components against *C. burnetii* in colostrum indirectly by giving a booster vaccination against *C. burnetii* to pregnant goats, as demonstrated in a vaccination trial of sows against porcine circovirus type 2 in pigs where the aim was to protect piglets from infection with this virus early in life [50]. Also, studies in which, pregnant animals were vaccinated using phase 1 *C. burnetii* vaccines have not reported any deleterious effects of the vaccine [22, 36].

In a dairy farm setting where new-born animals are restricted from receiving milk from their does, it seems reasonable to protect these animals as early as possible. However, considering the workload on intensive dairy farms during and immediately after kidding and the difficulty of ensuring that all kids receive adequate colostrum intake, we recommend vaccination be implemented not later than 8 weeks of age, i.e. preceding the majority of seroconversions in this study. We also recommend a booster dose to increase vaccine coverage, considering that antibodies derived from colostrum may still be present in some goats at 8 weeks of age and interfere with antibody production, even for inactivated vaccines like the Coxevac vaccine, (CEVA, France) used to vaccinate against *C. burnetii* in goats [51].

Cell-mediated immunity also plays an important role in protection against intra-cellular pathogens like *C. burnetii* and it is not affected by the presence of maternally derived antibodies, as demonstrated in a study that evaluated vaccination against Aujesky’s disease in pigs [51–53]; early vaccination, even in the presence of maternal antibody, may still be protective. Some studies have however, shown that some components of the immune system, for example, the B cells derived from the intestinal lymphoid tissues are not produced until after 8 weeks of age in goats [54]. Ideally the best time to vaccinate the kids would be during the narrow window of opportunity that occurs between the decline of the colostrum-derived maternal antibodies and the synthesis of kid-endogenous antibody arising from their environmental exposure to *C. burnetii*. This is probably from 8 to 10 weeks of age.

The comparable duration of seropositivity and median antibody titres of goats that seroconverted prior to and after the target breeding age suggests similarity in exposure before and after breeding. The duration of seropositivity varied widely, which accords with what has been previously reported in another outbreak in the UK [39]. The finding that the IgG phase 2 and 1 responses last longer than the IgM phase 2 and 1 responses that precede them is consistent with what has been reported in human serology and in adult animal studies [26, 46]. However, the number of secondary IgM responses compared to IgG responses, described in Table 2, may have been confounded by the lower repeatability of the IFA for IgM (78.1%) compared to IgG antibodies (96.9%) as well as the lower sensitivity of the IFA for IgM (88.8%) compared to IgG antibodies (94.8%).

The difference in risk associated with the two cohorts from two different kidding times studied here suggests that differences in the level of *C. burnetii* shedding at each kidding season, and possibly different levels of exposure to *C. burnetii* at birth, or soon after, may influence timing of occurrence of seroconversions against *C. burnetii* infections. This could also indicate differences in colostrum quality and its administration. Similarly, the surge in IgM and IgG seroconversions that occurred prior to weaning could possibly be due to exposure to *C. burnetii* at birth. The difference in the risk of seroconversion at farm level points to variability in contamination or shedding patterns on the different farms on the property; animals on Farm GS were partly reared by less intensive small out-grower farms until 4 months of age, which may explain the lower risk of seroconversion compared to Farm LC.

The increased risk of seroconversion in kids born to IgM seropositive does suggests recent infections in these does and points to either in utero transmission of *C. burnetii* or periparturient transmission. In utero transmission of *C. burnetii* has not been confirmed but *C. burnetii* DNA has been detected in amniotic fluid as well as spleen and kidneys of live and aborted kids; histopathological lesions similar to those in infected adult goats have also been reported in new-born goats [18, 36, 38]. Another study has also shown goat embryos to be highly susceptible to *C. burnetii* infection in vitro [55]. However, there is a challenge in differentiating DNA present because of *C. burnetii* infection from that due to contamination from the heavily infected placentas. The majority of the reports of PCR positive results from tissues of aborted kids provided very little information on how the prevention of contamination of these tissues by DNA from the heavily infected placental tissues was achieved [18, 36, 37]. Perhaps, the demonstration of vertical transmission of *C. burnetii* using PCR needs to be complemented by other methods, such as immunohistochemistry, that detect *C. burnetii* within the cells in tissues of foetuses or new-born kid goats.

As expected, exposure to *C. burnetii* shed at subsequent kidding seasons could be playing a role in increasing the proportion of infected goats before the target breeding age. This was more evident in cohort 2, where the rate of occurrence of antibody mediated responses was 4.5 times higher within than outside the kidding season. The comparable rate of occurrence of immune responses within and outside the kidding season for cohort 1 could be a result of a proportion of these goats being reared on out-grower farms for the first 4 months of their lives. The occurrence antibody-mediated immune responses outside the kidding seasons points to other risks of exposure to *C. burnetii* in the environment; for example, contaminated straw, hay or pastures, as well as inhalation or ingestion of contaminated dust particles especially during dry and windy weather [56–58].

Although occurrence of IgM and IgG responses and not the conventional twofold rise in IgG against phase 2 or IgM followed by IgG were used in comparing outside and within kidding season exposure to *C. burnetii*, this seemed the most practical way of computing animal-time at risk given the shorter duration of IgM antibodies compared to IgG as shown in Table 2. Comparisons for within and outside kidding seasons were also restricted to either IgM and IgG to cater for the differences in duration of IgM and IgG seropositivity as well as the differences in repeatability and sensitivity of the IFA for IgM and IgG antibodies. The detection of antibodies using the IFA may have been enhanced by repeated sampling and testing of samples in duplicate against IgG and IgM to phase 1 and 2 *C. burnetii* given that all IgG responses except one were preceded by IgM responses. The performance of the IFA may also have been enhanced by the quality of antigens used and the experience of the technicians in this instance, as these factors may influence the reproducibility of the test.

The shedding of *C. burnetii* by goats that seroconverted before breeding and remained seropositive through pregnancy underscores the role played by pre-breeding infections in the transmission of infection within the herd. Owing to mortality, we did not have a sufficiently large sample size at the end of the study to detect any statistically significant differences in *C. burnetii* shedding patterns between goats that seroconverted before breeding and those that seroconverted after breeding. Similarly, we were not able to detect any statistically significant reductions in weight gain at the individual animal level. These considerations are the focus of further ongoing studies. The high mortalities reaching up to 20% in the first months of follow-up were thought to result from a number of factors, including a poor-quality milk replacer and infectious causes like coccidiosis.

In summary, the first surge in the number of goats seroconverting with IgM antibodies to *C. burnetii* antigens was observed at 9 weeks of age, which underscores the need to vaccinate goats born on *C. burnetii* positive farms not later than 8 weeks of age. However, experimental studies are required to establish the effectiveness and feasibility of vaccinating goats at 8 weeks of age. It is expected that booster doses of vaccine will need to be administered to increase vaccine coverage. The shedding of *C. burnettii* by goats that remained seropositive after the initial seroconversion before the target breeding age provides more evidence supporting the notion that goats infected early in life can transmit *C. burnetii* to other goats and humans at their first kidding, underscoring the need to vaccinate young goats.

Post-weaning, the rate of seroconversion to *C. burnetii* was significantly higher within than outside the kidding seasons, this being more notable among goats in cohort 2, which were exposed to kidding season throughout the study period. Furthermore, goats from IgM seropositive does were two times more likely to seroconvert before the target breeding age, which points to either the occurrence of in utero transmission of *C. burnetii* or infection of goats during or shortly after birth.

Some of the goats that seroconverted before breeding shed *C. burnetii* at their first kidding, suggesting that goats infected early in life can be a risk for transmission of *C. burnetii* to susceptible animals in the herd.

No statistically significant reductions in weight gain at the individual animal level were observed among goats that seroconverted against *C. burnetii* before breeding.

## Additional files

**Additional file 1.** Kaplain-Meier survival curve showing the probability of intensively-reared kid goats remaining seronegative to *C. burnetii* before breeding, Victoria, Australia, 2015.

**Additional file 2.** Univariable assessment of the effect of risk factors on seroconversion against *C. burnetii* in goats.

**Additional file 3.** Multivariable Weibull accelerated failure time regression model assessing risk factors for seroconversion to *C. burnetii* before breeding in intensively-reared goats.

**Additional file 4.** Comparison of the rate of occurrence of antibody responses within and outside the kidding season in intensively managed goats.

**Additional file 5.** Median and inter-quartile range of antibody titres of goats in cohort 2 that shed *C. burnetii* at their first kidding during a Q fever outbreak farm.
