# Supplementary material for: A longitudinal study of serological responses to Coxiella burnetii and shedding at kidding among intensively-managed goats supports early use of vaccines
Source: Vet Res. 2017 Sep 15;48:50. doi: 10.1186/s13567-017-0452-3 (PMC5603018; doi:10.1186/s13567-017-0452-3)
Supplement: Supplementary file 2 — Additional file 2. Univariable assessment of the effect of risk factors on seroconversion against C. burnetii in goats. [file 13567_2017_452_MOESM2_ESM.docx]

### Additional file 2: Univariable assessment of the effect of risk factors on seroconversion against *C. burnetii* in goats

| Variables | Categories | Total | Events | Coef. | SE Coef. | P value | Survival rate ratio (95%CI) | Hazard ratio (95% CI) |
| --- | --- | --- | --- | --- | --- | --- | --- | --- |
| Farm | LC | 79 | 52 | -0.647 | 0.271 | 0.017 | 0.52 (0.31, 0.88) | 2.81 (1.19, 6.62) |
|  | GS | 16 | 6 | _ |  |  | 1.00 | 1.00 |
| Cohort | Cohort 2 | 62 | 42 | -0.483 | 0.181 | 0.007 | 0.62 (0.43, 0.88) | 2.19 (1.21, 3.98) |
|  | Cohort 1 | 33 | 16 | _ |  |  | 1.00 | 1.00 |
| Doe IgG | Positive | 48 | 30 | 0.144 | 0.176 | 0.413 | 1.16 (0.82, 1.63) | 0.80 (0.48, 1.36) |
|  | Negative | 45 | 27 | _ |  |  | 1.00 | 1.00 |
| Doe IgM | Positive | 37 | 29 | -0.309 | 0.178 | 0.083 | 0.73 (0.52, 1.04) | 1.59 (0.95, 2.67) |
|  | Negative | 56 | 28 | _ |  |  | 1.00 | 1.00 |
| Doe Parity | > 3 | 60 | 45 | 0.195 | 0.181 | 0.282 | 1.21 (0.85, 1.73) | 0.74 (0.43, 1.28) |
|  | ≤ 3 | 35 | 27 | _ |  |  | 1.00 | 1.00 |
| Colo. IgG P1 | Present | 71 | 51 | 0.143 | 0.276 | 0.604 | 1.15 (0.67, 1.98) | 0.81 (0.37, 1.79) |
|  | Absent | 9 | 7 | _ |  |  | 1.00 | 1.00 |
| Colo. IgG P2 | Present | 72 | 53 | -0.375 | 0.310 | 0.227 | 0.69 (0.37, 1.26) | 1.76 (0.70, 4.44) |
|  | Absent | 8 | 5 | _ |  |  | 1.00 | 1.00 |
| Colo. IgG P1 | ≥640 | 52 | 41 | -0.124 | 0.210 | 0.554 | 0.88 (0.59, 1.33) | 1.20 (0.66, 2.16) |
|  | <640 | 21 | 15 | _ |  |  | 1.00 | 1.00 |
| Colo  IgG P1 | ≥320 | 58 | 47 | -0.453 | 0.248 | 0.068 | 0.64 (0.39, 1.03) | 1.95 (0.95, 3.99) |
|  | <320 | 15 | 9 | _ |  |  | 1.00 | 1.00 |
| Colo.  IgG P2 | ≥640 | 55 | 44 | -0.380 | 0.220 | 0.084 | 0.68 (0.44, 1.05) | 1.76 (0.92, 3.35) |
|  | <640 | 19 | 12 | _ |  |  |  | 1.00 |
| Colo.  IgG P2 | ≥320 | 59 | 45 | -0.138 | 0.233 | 0.555 | 0.87 (0.55, 1.38) | 1.22 (0.63, 2.36) |
|  | <320 | 15 | 11 | _ |  |  | 1.00 | 1.00 |
| Colo.  Duration | >4weeks | 51 | 40 | -0.275 | 0.200 | 0.168 | 0.76 (0.51, 1.12) | 1.50 (0.85, 2.64) |
|  | ≤4weeks | 20 | 16 | _ |  |  | 1.00 | 1.00 |

Colo. = maternally-derived colostrum antibodies detected in the kid’s sera. Coef. = Coefficient, SE = standard error, CI is confidence interval. The farm, the cohort, IgM status of the source doe, having colostrum IgG phase 1 antibodies titres ≥320 and IgG phase 2 colostrum titres ≥640 was associated with a higher probability of not seroconverting against *C. burnetii* before 28 weeks of age in goats. The intercepts were removed from the univariate models to ease presentation.
