# Supplementary material for: A longitudinal study of serological responses to Coxiella burnetii and shedding at kidding among intensively-managed goats supports early use of vaccines
Source: Vet Res. 2017 Sep 15;48:50. doi: 10.1186/s13567-017-0452-3 (PMC5603018; doi:10.1186/s13567-017-0452-3)
Supplement: Supplementary file 3 — Additional file 3. Multivariable Weibull accelerated failure time regression model assessing risk factors for seroconversion to C. burnetii before breeding in intensively-reared goats. [file 13567_2017_452_MOESM3_ESM.docx]

**Additional file 3: Multivariable Weibull accelerated failure time regression model assessing risk factors for seroconversion to *C. burnetii* before breeding in intensively-reared goats**

| Variable | Levels | n | Sero. | Coef. | SE (Coef.) | P value | Survival rate ratio (95% CI) | Hazard ratio (95% CI) |
| --- | --- | --- | --- | --- | --- | --- | --- | --- |
| Farm | LC | 79 | 52 | -0.837 | 0.266 | 0.002 | 0.43 (0.23, 0.73) | 4.04 (1.65, 9.92) |
|  | GS | 16 | 6 |  |  |  | 1.00 | 1.00 |
| Doe IgM | Positive | 37 | 29 | -0.481 | 0.163 | 0.003 | 0.62 (0.45, 0.85) | 2.23 (1.29, 3.86) |
|  | Negative | 56 | 28 |  |  |  | 1.00 | 1.00 |
| Intercept | – | – | – | 4.477 | 0.536 | <0.001 | 87.94 (30.78, 251.23) | – |

Interpretation: After adjusting for the effect of farm, kids born by does that had positive IgM titres (Doe IgM = positive; indicating recent exposure) were 2.23 times more likely to seroconvert within the first 6 months of life compared to those born by IgM seronegative does. Log likelihood = -200.4. Coef. = coefficient. Sero. = seroconverted.
