# Supplementary material for: A longitudinal study of serological responses to Coxiella burnetii and shedding at kidding among intensively-managed goats supports early use of vaccines
Source: Vet Res. 2017 Sep 15;48:50. doi: 10.1186/s13567-017-0452-3 (PMC5603018; doi:10.1186/s13567-017-0452-3)
Supplement: Supplementary file 4 — Additional file 4. Comparison of the rate of occurrence of antibody responses within and outside the kidding season in intensively managed goats. [file 13567_2017_452_MOESM4_ESM.docx]

**Additional file 4: Comparison of the rate of occurrence of antibody responses within and outside the kidding season in intensively managed goats**

| Cohort | Antibody  Type | Kidding season | Goat weeks at risk | Number of antibody responses | Rate of occurrence of antibody responses (per 100 goat weeks at risk) | Occurrence rate ratio (95% CI) |
| --- | --- | --- | --- | --- | --- | --- |
| 1 | IgM | Within | 224.25 | 18 | 8.0 (4.8, 12.7) | 1.4 (0.71, 2.7) |
|  |  | Outside | 290.75 | 17 | 3.4 (2.2, 4.8) |  |
|  | IgG | Within | 261.25 | 11 | 4.2 (2.1, 7.5) | 1.3 (0.5, 3.0) |
|  |  | Outside | 298.75 | 10 | 3.3 (1.6, 6.2) |  |
| 2 | IgM | Within | 323.50 | 28 | 8.7(5.8, 12.5) | 4.1 (2.1, 8.2) |
|  |  | Outside | 575.00 | 12 | 2.1 (1.1, 3.6) |  |
|  | IgG | Within | 218.00 | 34 | 15.6 (10.8, 21.8) | 4.5 (2.1, 9.8) |
|  |  | Outside | 232.50 | 8 | 3.4 (1.4, 6.8) |  |

The rate of occurrence of antibody responses to *C. burnetii* was four times higher within the kidding season than outside the kidding season among goats in cohort 2. However, the rate of occurrence of antibody responses was not statistically different within the kidding season and outside the kidding season.
