# Supplementary material for: A longitudinal study of serological responses to Coxiella burnetii and shedding at kidding among intensively-managed goats supports early use of vaccines
Source: Vet Res. 2017 Sep 15;48:50. doi: 10.1186/s13567-017-0452-3 (PMC5603018; doi:10.1186/s13567-017-0452-3)
Supplement: Supplementary file 4 [file 13567_2017_452_MOESM1_ESM.docx]

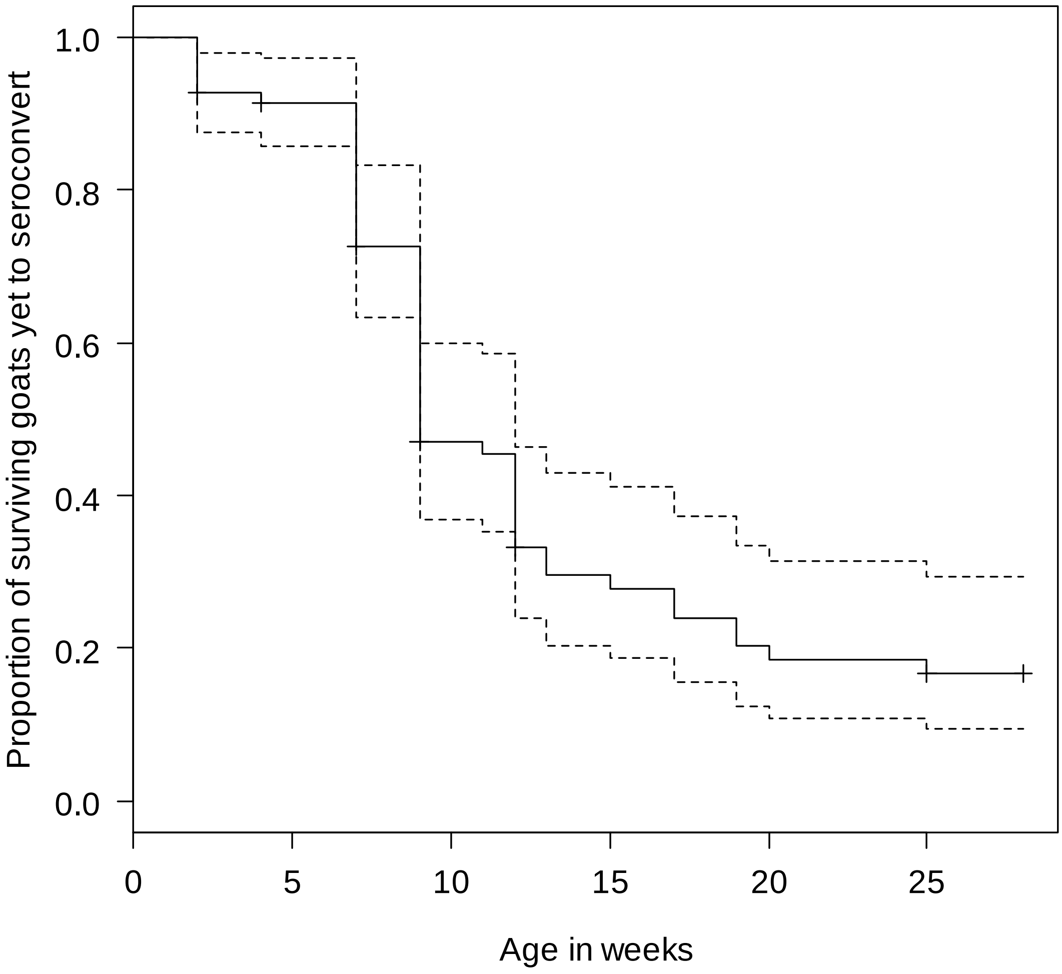

Proportion of surviving goats yet to seroconvert
1.0
0.8
0.6
0.4
0.2
0.0
0
5
10
15
20
25
Age in weeks
