# Supplementary material for: A longitudinal study of serological responses to Coxiella burnetii and shedding at kidding among intensively-managed goats supports early use of vaccines
Source: Vet Res. 2017 Sep 15;48:50. doi: 10.1186/s13567-017-0452-3 (PMC5603018; doi:10.1186/s13567-017-0452-3)
Supplement: Supplementary file 5 [file 13567_2017_452_MOESM5_ESM.docx]

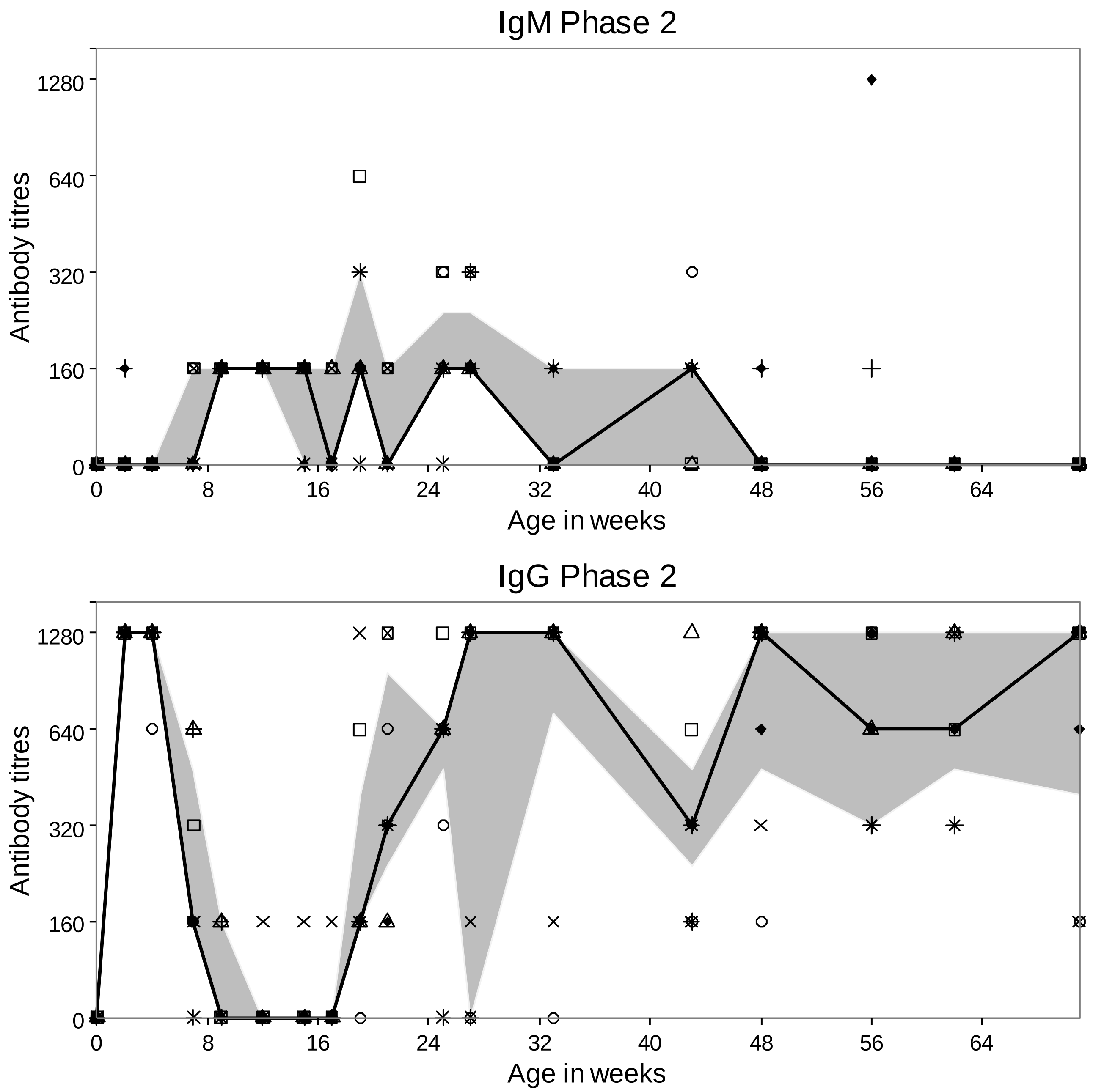
IgM Phase 2
Antibody titres
1280
640
320
160
0
0
8
16
24
32
40
48
56
64
Age in weeks
IgG Phase 2
Antibody titres
1280
640
320
160
0
0
8
16
24
32
40
48
56
64
Age in weeks
